# Delving deep into the draft genome of Mangrovibacter sp. SLW1, isolated from Sundarbans mangrove

**DOI:** 10.1099/acmi.0.000847.v3

**Published:** 2024-08-21

**Authors:** Arindam Roy, Anwesha Ghosh, Punyasloke Bhadury

**Affiliations:** 1 Integrative Taxonomy and Microbial Ecology Research Group, Department of Biological Sciences, Indian Institute of Science Education and Research Kolkata, Mohanpur, Nadia, 741246, West Bengal, India; 2 Centre for Climate and Environmental Studies, Indian Institute of Science Education and Research Kolkata, Mohanpur, Nadia, 741246, West Bengal, India

**Keywords:** carbohydrates, heavy metals, mangrove, *Mangrovibacter*, siderophore

## Abstract

*Mangrovibacter* sp. SLW1, a Gram-negative, aerobic, motile bacterium, was isolated from mangrove litterfall in Sundarbans mangrove. The draft genome is 5.5 Mbp in size with 49.45 mol% guanine-cytosine (GC) content. The linear chromosome of the bacterium consists of 27 contigs with 7339 coding sequences. The detailed *in silico* analyses of the genome of *Mangrovibacter* sp. SLW1 provide information on ecological adaptation. The genome is a reservoir for multiple heavy metals and metalloid resistance gene clusters as well as exhibit metabolic capabilities for utilization of a wide range of carbohydrates. It also encodes for tris-catecholate siderophore and can regulate uptake of iron thereby may influence plant growth such as mangrove vegetation.

## Data Summary

The whole-genome sequence data reported in this manuscript is available in NCBI as BioProject accession number PRJNA1108486 and BioSample accession number SAMN41243833. All other data is included within this article or in the associated supplementary files.

## Announcement

**Fig. 1.**
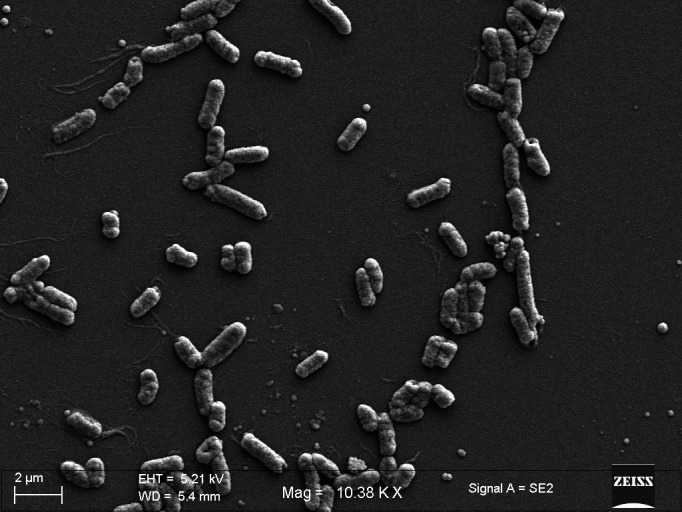
Field emission scanning electron microscopy image of *Mangrovibacter* sp. SLW1; scale bar at the left bottom side.

**Fig. 2.**
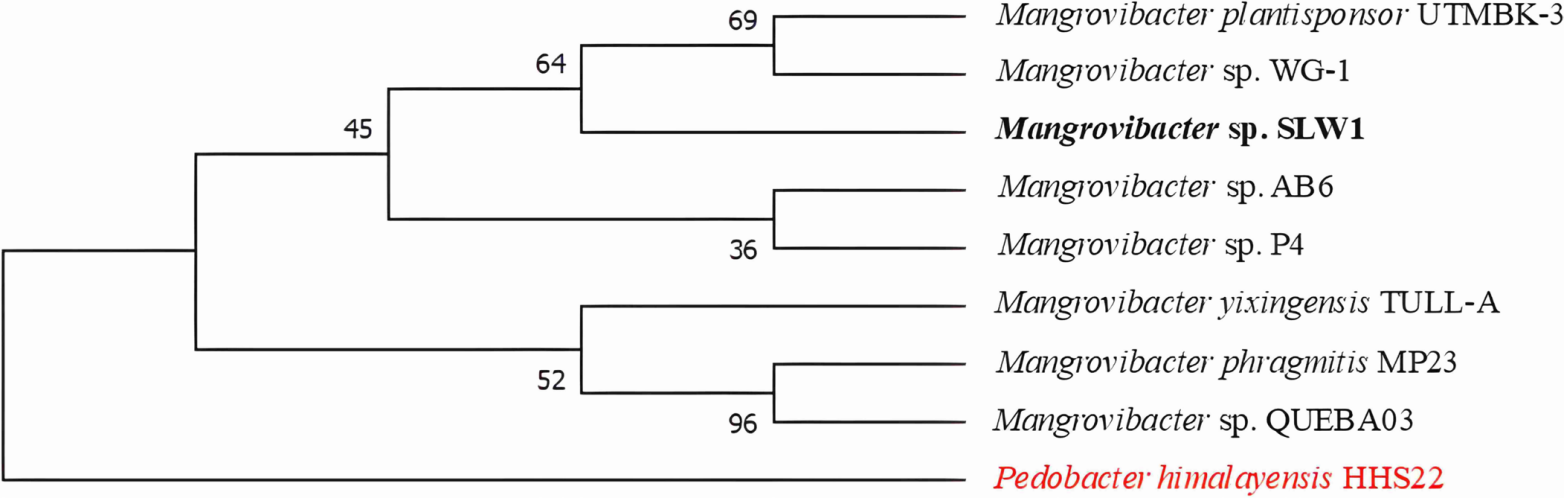
Phylogenetic analysis based on 16S rRNA sequence from maximum likelihood–based phylogenetic tree with Hasegawa–Kishino–Yano model (1000 bootstrap replicates; bold font marks investigated strain, and red font indicates out-group).

Mangrove sediments harbour a wide array of functional bacterial groups modulating key ecosystem processes. Members of the genus *Mangrovibacter*, belonging to the family Enterobacteriaceae, are ubiquitous across coastal ecosystems globally and augment microbially mediated biogeochemistry. Previously reported *Mangrovibacter* strains provide ecological cues to multiple nutrient and environmental stressors due to their diversified metabolite utilization strategies, i.e. nitrogen fixation, making them ideal candidates for plant growth regulation [1,3]. The bacterium reported in the present study was isolated from ‘true’ mangrove *Avicennia alba* litterfall, collected in July 2023 from Stn3 of Sundarbans Biological Observatory Time Series (SBOTS) in Sagar Island of the Sundarbans (India) as part of coastal biodiscovery initiative. The isolate was grown in tannic acid agar medium (0.5 % w/v) with salinity of 20 and pH 7.6 and subsequently cultured on Luria–Bertani broth with the same parameters for genomic DNA (gDNA) extraction. The gDNA was extracted by using modified published protocol [4,5]. The quality of gDNA was visualized on 1 % agarose gel electrophoresis and quantified using a NanoDrop 2000c Spectrophotometer (Thermo Fisher Scientific, USA). The whole-genome sequencing was undertaken in a MinION platform using Oxford Nanopore sequencing chemistry. The library was prepared using ligation sequencing kit (SQK-LSK109, Oxford Nanopore Technologies, UK). The library was purified using magnetic beads (Sergi Lab Supplies LLC, USA) and subsequently loaded on R9.4.1 flowcell for basecalling. The quality check for downstream analyses of generated fastq files was set at *Q*>8. Adapters and low-quality reads were removed using porechop (v0.2.0) and fittlong (v0.2.1). The *de novo* genome assembly for filtered long reads were performed using flye (v2.9.3 [6]). Assembled genome quality was evaluated through application of QUAST package (v5.2.0 [7]) and annotated using Prokka (v1.14.6 [8]). Genome completeness was checked using CheckM (v1.0.18 [9]). In brief, overnight grown culture was harvested at 5000 r.p.m., washed using phosphate-buffered saline (PBS) (pH 7.4) and kept in 2.5 % glutaraldehyde PBS solution for overnight. Subsequently, the culture was treated with 1 % osmium tetroxide followed by dehydration with gradation of ethanol. Finally, cells were drop casted, dried, gold:palladium coated (20 : 80) and observed under Carl Zeiss Sigma Field Emission Scanning Electron Microscope (FESEM). *In silico* phenotyping was carried out by traitar [10] and genomeSPOT tools [11]. Prediction of carbohydrate-active enzymes and putative substrate annotation was performed using dbCAN3 server [12]. CRISPR-Cas arrays were searched in the assembled genome by CRISPR-Cas Finder [13]. The presence of biosynthetic gene clusters (BGCs), ribosomally synthesized and post-translationally modified peptides, was detected by antiSMASH 6.1.1 [14] and BAGEL4 server [15], respectively. Antimicrobial resistance genes (ARGs) were annotated and identified through the resistance gene identifier (RGI) module of Comprehensive Antibiotic Resistance Database [16]. Metal and metalloid resistance genes and gene clusters were identified by DeepMRG [17]. The circular genome map was visualized in Proksee [18]. The phylogenomic analysis for the isolated strain was undertaken in Type Strain Genome Server [19]. Genome BLAST distance phylogeny was calculated using genome data, and phylogenomic tree was constructed using FastME (v2.0 [20]). Digital DNA–DNA hybridization, genome-to-genome distance calculator (GGDC v3.0) and average nucleotide identity (ANI) were calculated using the DSMZ server [21] and OrthoANI [22], respectively. The genome sequencing of strain SLW1 yielded a total of 84 916 raw reads with 325 123 327 bp, and the circular genome map is included (Fig. S1, available in the online Supplementary Material). The *de novo* assembly of filtered raw reads provided a clear resolution of 27 contigs, covering a length of 5 533 946 bp. The G+C content is 49.45 % with the largest contig of 3 259 965 bp. The assembly quality statistics and genomic features are listed in Table 1. The draft genome sequence comprises of 84 transfer RNAs, 22 ribosomal RNAs (rRNAs) and 7339 coding sequences. Genome annotation also revealed the presence of 2769 hypothetical protein sequences. The genome encodes for flagellar biosynthesis protein (*FlhA*, *Flhb* and *FliR*), basal body rod protein (*FlgB* and *FlgC*), assembly protein (*FliH*), motor switch protein and L, M and P ring protein, which supported the evidence of motility in wet lab experiment as well as based on FESEM (Fig. 1). CheckM revealed 94.37 % completeness of genome. The 16S rRNA–based phylogenetic analysis assigned the strain SLW1 as *Mangrovibacter* sp. and forms cluster with *Mangrovibacter plantisponsor* (Fig. 2). The GGDC for related *Mangrovibacter* strain showed the minimum distance of 0.0070 with *Mangrovibacter yixingensis*. DNA–DNA hybridization and ANI values also underpin the strain SLW1 close affiliation with *M. yixingensis* (Table S1, available in the online Supplementary Material). The genome based phylogeny also reflected the taxonomic affiliation of SLW1 (Fig S2, available in the online Supplementary Material). GenomeSPOT *in silico* phenotyping for growth parameters predicts that *Mangrovibacter* sp. SLW1 can grow in pH ranging between 4.74 and 9.32 whereas optimum temperature for growth is projected at 24.9 ˚C. The maximum salinity tolerance level is 6.18 (% w/v) NaCl. The phypat+PGL predictor also revealed the ability of this isolate to utilize acetate and towards production of beta-galactosidase. The isolate is catalase positive indicating the adaptation for environmental stress tolerance [23]. Based on the prediction of CAZy database and strict usage of the three identification tools, i.e. DIAMOND:CAZy, HMMER:dbCAN and HMMER:dbCAN-sub, default parameters revealed the presence of genes from 21 different families of glycoside hydrolases, 2 different families of carbohydrate esterases and 14 different families of glycosyltransferases. The studied genome also encodes for genes from four different families of polysaccharide lyases. The enzymes from glycoside hydrolase family, i.e. GH1, GH2, GH3, GH42 and GH43, are responsible for complex carbohydrate degradation [24]. Traitar phenotyping also predicts for the catabolism of a wide range of growth substrates, i.e. myo-inositol, d-xylose, cellobiose, d-mannitol, trehalose, maltose, melibiose, d-mannose, l-arabinose, lactose, raffinose, rhamnose and sucrose. The source of complex carbon in mangrove ecosystem can be explained by the high carbohydrate content in the leaves and roots [25]. Substrate annotation by dbCAN3 provides insights into the following substrate utilization, i.e. pectin, starch, cellulose, galactan and alginate by *Mangrovibacter* sp. SLW1. Cleaving of oligosaccharides by GH42 family encoding genes leads to the generation of monosaccharides [26], promotes growth of other microorganisms and contributes towards the labile organic carbon pool. The genome of isolated strain possess two CRISPR arrays at contig 11 of 1 585 bp and 694 bp containing 26 and 11 spacers, respectively, with conserved direct repeats length of 28 for each one (Table S2, available in the online Supplementary Material). The presence of CRISPR-Cas system with higher number spacers possibly plays a role in the survival strategy of *Mangrovibacter* sp. SLW1 to combat biotic stressors [27]. Apart from this, CRISPR Finder prediction also revealed the presence of seven Cas gene clusters cas1_TypeIE, cas5_TypeIE, cas5_TypeI, cas7_TypeIE, cas6_TypeIE, cse2_TypeIE and cas3a_TypeI. BGC annotation using BAGEL4 showed the presence of gene cluster for antimicrobial compound bottromycin. The antiSMASH 6.1.1 predicted non-ribosomal peptide synthetase-like (NRPS-like) BGCs (53 %) and thiopeptide BGC with less (<40 %) homology on contig 11. The remaining 25 contigs do not have any BGCs. The *in silico* analysis also suggested the presence of tris-catecholate siderophore turnerbactin, chrysobactin, dichrysobactin, trichrysobactin and enterobactin secondary metabolites in NRPS-like BGCs. The presence of enterobactin ABC transporter system coupled with iron (*fepB*, *fepG* and *fepD*) plays a putative role in iron uptake [28] and assimilation of iron in Sundarbans mangrove vegetation, modulating plant growth. The genome has the presence of partial pathways for biosynthesis of a range of secondary metabolites including betalains, carbapenems and isoquinoline. The enzyme type II PKS ketosynthase, involved in aurachin biosynthesis, is also present. The resistome of isolated bacterium has *emrR*, *rsmA*, CRP, H-NS and *qacG* as predicted using RGI database (strict hits only). The presence of antibiotic efflux pump mechanism confers resistance to multiple classes of antibiotics, i.e. fluoroquinolones, diaminopyrimidine, macrolides, penems, cephalosporin, cephamycin and tetramycin. Resistance to few antibiotic drug classes can also be attributed to target alteration. The multiple carbon source utilization mechanism leads to the acquisition of less ARGs [29]. Genome-wide analysis indicates that *Mangrovibacter* sp. SLW1 hosts a repertoire of metal and metalloid resistance genes and gene clusters towards nickel, zinc, cadmium, tungsten, arsenic, antimony, copper, tellurium, cobalt, magnesium, manganese, molybdenum, iron, lead, selenium, mercury, gallium, chromium and gallium (Table S3, available in the online Supplementary Material). It carries genes for translocating P-type ATPases which confer resistance to Zn^2+^, Cd^2+^, Pb^2+^ and Cu^2+^ [30]. The presence of P-type ATPases is crucial for transportation of heavy metals from cytoplasm to periplasm, thereby maintaining ion homeostasis. *mntH* gene encodes Cd^2+^ uptake, facilitating cadmium efflux from cells. The genome also carries gene for arsenate reductase (*arsC*), mediating the transformation from As^5+^ to As^3+^ [31]. Copper resistance proteins A, B and C act as key factors in copper resistance. Also, the presence of ABC transporter ATP-binding protein (*yadG*) putatively indicates a role in copper uptake required for cellular metabolism. DeepMRG annotation also predicts the presence of *corC* gene, a magnesium and cobalt efflux protein responsible for Co^2+^ resistance, whereas *rcnB* gene encodes for nickel–cobalt ion homeostasis. A wide spectrum of metal resistance genes in the genome of *Mangrovibacter* sp. SLW1 explained the collection site as a potential sink of heavy metals and most likely reeling from anthropogenic stressors. KEGG annotation revealed the presence of 40.3 % metabolic pathways in strain SLW1. Environmental information processing genes and carbohydrate metabolism-related genes accounted for 21.88 %, revealing the ecological signals on local scale. The genome encodes for nitrilase (EC 3.5.5.1), 4-carboxymuconolactone decarboxylase (EC 4.1.1.44), acetyl CoA acyltransferase (EC 2.3.1.16), acetyl dehydrogenase (EC 1.2.1.10), 4-oxalocrotonate tautomerase (EC 5.3.2.6) and 4-oxalmesaconate hydratase (EC 4.2.1.83), putatively indicating the ability to break down benzoate compound. The KEGG annotation also focused on enzymatic pathway for aminobenzoate metabolism, (S)-mandelate dehydrogenase (EC 1.1.99.31), 4-hydroxybenzoate decarboxylase subunit C (EC 4.1.1.61), gallate dioxygenase (EC 1.13.11.57) and gallate decarboxylase subunit C (EC 4.1.1.59). The strain also possess genetic information linked to naphthalene, styrene, toluene, xylene and atrazine degradation. Such broad range of xenobiotics breakdown indicated that the Sundarbans mangrove is possibly becoming a sink of pollutants of anthropogenic origin. In this study, the genomic description of *Mangrovibacter* sp. SLW1 revealed the presence of a plethora of metal and metalloid resistance genes, BGCs and shed light on the metabolic capability to degrade aromatic compounds. This study also paves the way to further investigate the strain as a potential biomonitoring tool for long-term tracking of ecological health of coastal ecosystems.

**Table 1.** Draft genome statistics of *Mangrovibacter* sp. SLW1

Genomic features	No. or length (bp)
G+C content (%)*	49.45
No. of all contigs	27
No. of large contigs (>1000 bp)	27
Contig N50	3 259 965
Contig N90	838 191
L50	1
L90	3
Total length	5 533 946

G+C content- guanine +cytosine

## supplementary material

10.1099/acmi.0.000847.v3 Uncited Supplementary Material 1.

## Abbreviations

BGC: biosynthetic gene cluster
gDNA: genomic DNA
GGDC: genome-to-genome distance calculator
NRPS: non-ribosomal peptide synthetase
PBS: phosphate-buffered saline
RGI: resistance gene identifier
rRNA: ribosomal RNA
