# Supplementary material for: Delving deep into the draft genome of Mangrovibacter sp. SLW1, isolated from Sundarbans mangrove
Source: Access Microbiol. 2024 Aug 21;6(8):000847.v3. doi: 10.1099/acmi.0.000847.v3 (PMC11652732; doi:10.1099/acmi.0.000847.v3)

Supplementary Materials

Table S1: Comparison of *Mangrovibacter* sp. SLW1 with three closely related species

Species	OrthoANIu (%)	GGDC	DNA-DNA relatedness(%)	G+C (%)
Mangrovibacter yixingensis	99.39	0.0070	94.7	52
Mangrovibacter plantisponsor	89.77	0.1029	39	50.43
Mangrovibacter phragmitis	88.67	0.1152	35.7	49.91

Table S2: CRISPR Cas arrays in *Mangrovibacter* sp. SLW1

Contig	Start	End	Repeat consensus	Spacer	Evidence level
Contig_11	652645	654230	TTTCTAAGCTGCCTGTACGGCAGCGAAC	26	4
Contig_11	1415258	1415952	GAAACACCCCCACGTACGTGGGGAAGAC	11	4

Table S3: Heavy metal and metalloid resistome

Proteins	Metal and metalloids
Multidrug efflux ATP-binding/permease protein	Nickel (Ni)
Multiple antibiotic resistance protein MarA	Zinc (Zn)
Transcriptional activator protein CopR	Cadmium (Cd),Zinc (Zn)
Sensor histidine kinase RcsC	Tungsten (W),Zinc (Zn)
HTH-type transcriptional activator RhaS	Zinc (Zn)
Maltose/maltodextrin import ATP-binding protein MalK	Arsenic (As)
HTH-type transcriptional regulator CueR	Copper (Cu)
putative iron export permease protein FetB	Iron (Fe)
Cobalt import ATP-binding protein CbiO	Iron (Fe)
Propanediol diffusion facilitator	Antimony (Sb),Arsenic (As)
hypothetical protein	Cobalt (Co),Magnesium (Mg)
Multidrug resistance protein MdtA	Zinc (Zn)
Multidrug resistance protein MdtB	Zinc (Zn)
Multidrug resistance protein MdtC	Zinc (Zn)
Signal transduction histidine-protein kinase BaeS	Tungsten (W),Zinc (Zn)
Transcriptional regulatory protein BaeR	Tungsten (W),Zinc (Zn)
Aliphatic sulfonates import ATP-binding protein SsuB	Tungsten (W)
Vitamin B12 import ATP-binding protein BtuD	Tungsten (W)
Glycine betaine uptake system ATP-binding protein YehX	Tungsten (W)
Galactose/methyl galactoside import ATP-binding protein MglA	Iron (Fe),Manganese (Mn)
Galactose/methyl galactoside import ATP-binding protein MglA	Tungsten (W)
Vitamin B12 import system permease protein BtuC	Cobalt (Co),Nickel (Ni)
Inner membrane ABC transporter permease protein YejE	Nickel (Ni)
putative efflux pump outer membrane protein TtgC	Copper (Cu)

Divalent metal cation transporter MntH	Cadmium (Cd),Cobalt (Co),Iron (Fe),Manganese (Mn),Zinc (Zn)
Sulfate/thiosulfate import ATP-binding protein CysA	Tungsten (W)
Sulfate transport system permease protein CysW	Molybdenum (Mo),Tungsten (W)
Sulfate transport system permease protein CysT	Molybdenum (Mo),Tungsten (W)
putative protein YfgD	Antimony (Sb),Arsenic (As)
hypothetical protein	Zinc (Zn)
Sensor histidine kinase QseE	Iron (Fe)
hypothetical protein	Cobalt (Co),Magnesium (Mg)
Putative two-component membrane permease complex subunit SMU_747c	Arsenic (As)
Nickel/cobalt homeostasis protein RcnB	Cobalt (Co),Nickel (Ni)
Toluene efflux pump outer membrane protein TtgI	Copper (Cu)
Diacetylchitobiose uptake system ATP-binding protein MsiK	Arsenic (As)
Outer membrane protein OprM	Copper (Cu),Silver (Ag)
Enterobactin exporter EntS	Cobalt (Co),Nickel (Ni)
Thiol:disulfide interchange protein DsbC	Copper (Cu)
Multiple stress resistance protein BhsA	Copper (Cu)
Sulfate/thiosulfate import ATP-binding protein CysA	Tungsten (W)
Cell division protein FtsP	Copper (Cu)
Glutamine transport ATP-binding protein GlnQ	Tungsten (W)
Zinc transporter ZupT	Cadmium (Cd),Cobalt (Co),Copper (Cu),Iron (Fe),Nickel (Ni),Zinc (Zn)
NADPH-dependent ferric-chelate reductase	Iron (Fe),Nickel (Ni)
Vitamin B12 import ATP-binding protein BtuD	Nickel (Ni)
Glutathione transport system permease protein GsiD	Nickel (Ni)
Glutathione transport system permease protein GsiC	Nickel (Ni)
putative D,D-dipeptide-binding periplasmic protein DdpA	Nickel (Ni)
Dipeptide transport system permease protein DppB	Nickel (Ni)
putative D,D-dipeptide transport system permease protein DdpC	Nickel (Ni)

Putative membrane-bound redox modulator Alx	Tellurium (Te)
Transcriptional regulatory protein BasR	Iron (Fe)
Sensor protein BasS	Iron (Fe)
Lipopolysaccharide export system ATP-binding protein LptB	Molybdenum (Mo),Tungsten (W)
putative protein McbA	Cadmium (Cd)
HTH-type transcriptional regulator ZntR	Zinc (Zn)
Bacterioferritin	Iron (Fe)
Phosphate-binding protein PstS	Arsenic (As)
Transcriptional regulatory protein OmpR	Zinc (Zn)
sn-glycerol-3-phosphate import ATP-binding protein UgpC	Molybdenum (Mo),Tungsten (W)
Zinc/cadmium/lead-transporting P-type ATPase	Cadmium (Cd),Lead (Pb),Zinc (Zn)
Transcriptional repressor FrmR	Cobalt (Co),Nickel (Ni)
Dipeptide transport system permease protein DppC	Nickel (Ni)
Dipeptide transport system permease protein DppB	Nickel (Ni)
Superoxide dismutase [Mn]	Zinc (Zn)
HTH-type transcriptional activator RhaS	Selenium (Se)
Sulfate/thiosulfate import ATP-binding protein CysA	Zinc (Zn)
Formate dehydrogenase-O iron-sulfur subunit	Tungsten (W)
Arsenate reductase	Arsenic (As)
Arsenical pump membrane protein	Antimony (Sb),Arsenic (As)
hypothetical protein	Antimony (Sb),Arsenic (As)
Ribose import ATP-binding protein RbsA	Antimony (Sb),Arsenic (As)
Galactose/methyl galactoside import ATP-binding protein MglA	Iron (Fe)
hypothetical protein	Molybdenum (Mo),Tungsten (W)
hypothetical protein	Copper (Cu)
Phosphate import ATP-binding protein PstB	Molybdenum (Mo),Tungsten (W)
Phosphate transport system permease protein PstA	Arsenic (As)

Phosphate transport system permease protein PstC	Arsenic (As)
Phosphate-binding protein PstS	Arsenic (As)
Ribose import ATP-binding protein RbsA	Arsenic (As)
Thiol:disulfide interchange protein DsbA	Tungsten (W)
Dimethyl sulfoxide reductase DmsA	Cadmium (Cd),Mercury (Hg),Zinc (Zn)
Anaerobic dimethyl sulfoxide reductase chain B	Arsenic (As)
Transcriptional regulatory protein CpxR	Arsenic (As)
Ferrous-iron efflux pump FieF	Zinc (Zn)
Glycerol uptake facilitator protein	Cadmium (Cd),Cobalt (Co),Iron (Fe),Nickel (Ni),Zinc (Zn)
Magnesium transport protein CorA	Antimony (Sb),Arsenic (As)
Zinc uptake regulation protein	Cobalt (Co),Magnesium (Mg),Manganese (Mn),Nickel (Ni)
Manganese transport system membrane protein MntB	Zinc (Zn)
Manganese import ATP-binding protein ScaC	Iron (Fe),Manganese (Mn),Zinc (Zn)
Sodium/glucose cotransporter	Manganese (Mn)
Regulatory protein SoxS	Tellurium (Te)
Cation/acetate symporter ActP	Zinc (Zn)
Ferrichrome receptor FcuA	Tellurium (Te)
Alpha-D-ribose 1-methylphosphonate 5-triphosphate synthase subunit PhnL	Cadmium (Cd),Cobalt (Co),Copper (Cu),Gallium (Ga),Iron (Fe),Manganese (Mn),Nickel (Ni),Zinc (Zn)
Cation efflux system protein CusB	Nickel (Ni)
Cation efflux system protein CusA	Silver (Ag)
Glutathione transport system permease protein GsiC	Silver (Ag)
Glutathione transport system permease protein GsiD	Nickel (Ni)
hypothetical protein	Nickel (Ni)
Biofilm growth-associated repressor	Zinc (Zn)
Divalent-cation tolerance protein CutA	Zinc (Zn)
Glutathione transport system permease protein GsiC	Copper (Cu)

Glutathione transport system permease protein GsiD	Nickel (Ni)
Right origin-binding protein	Nickel (Ni)
Aerobic respiration control protein ArcA	Cadmium (Cd),Mercury (Hg),Silver (Ag)
Protein ApaG	Zinc (Zn)
Thiamine import ATP-binding protein ThiQ	Cobalt (Co),Magnesium (Mg)
Dihydrolipoyl dehydrogenase	Molybdenum (Mo),Tungsten (W)
Blue copper oxidase CueO	Mercury (Hg)
putative ABC transporter ATP-binding protein YadG	Copper (Cu)
Iron(3+)-hydroxamate import system permease protein FhuB	Tungsten (W)
Lipoprotein NlpE	Cobalt (Co),Nickel (Ni)
Methionine import ATP-binding protein MetN	Copper (Cu)
Formate dehydrogenase-O iron-sulfur subunit	Tungsten (W)
hypothetical protein	Arsenic (As)
Bacterial non-heme ferritin	Cadmium (Cd)
Copper homeostasis protein CutC	Copper (Cu),Iron (Fe),Manganese (Mn)
Holliday junction ATP-dependent DNA helicase RuvB	Copper (Cu)
High-affinity zinc uptake system membrane protein ZnuB	Chromium (Cr),Selenium (Se),Tellurium (Te)
High-affinity zinc uptake system protein ZnuA	Zinc (Zn)
putative manganese efflux pump MntP	Zinc (Zn)
Multidrug resistance protein MdtB	Magnesium (Mg),Manganese (Mn)
Toluene efflux pump periplasmic linker protein TtgG	Cobalt (Co),Nickel (Ni)
Calcium-transporting ATPase	Zinc (Zn)
Periplasmic dipeptide transport protein	Cobalt (Co)
hypothetical protein	Nickel (Ni)
Oligopeptide transport system permease protein OppB	Cadmium (Cd)
Aconitate hydratase A	Nickel (Ni)
Peptide transport system permease protein SapC	Iron (Fe)

Peptide transport periplasmic protein SapA	Nickel (Ni)
Psp operon transcriptional activator	Nickel (Ni)
tRNA 5-carboxymethoxyuridine methyltransferase	Zinc (Zn)
Transposon Tn10 TetD protein	Tellurium (Te)
Transcriptional repressor RcnR	Zinc (Zn)
hypothetical protein	Cobalt (Co),Iron (Fe),Nickel (Ni)
Spermidine/putrescine import ATP-binding protein PotA	Nickel (Ni)
Inner membrane ABC transporter permease protein YdcU	Molybdenum (Mo),Tungsten (W)
Inner membrane ABC transporter permease protein YdcV	Molybdenum (Mo),Tungsten (W)
Bicarbonate transport ATP-binding protein CmpD	Tungsten (W)
Metal-pseudopaline receptor CntO	Cobalt (Co),Copper (Cu),Gallium (Ga),Iron (Fe),Nickel (Ni)
Multiple antibiotic resistance protein MarA	Zinc (Zn)
Sensor histidine kinase RcsC	Tungsten (W),Zinc (Zn)
hypothetical protein	Molybdenum (Mo),Tungsten (W)
Putative dimethyl sulfoxide reductase chain YnfE	Arsenic (As)
Anaerobic dimethyl sulfoxide reductase chain B	Arsenic (As)
Transcriptional regulatory protein RstA	Zinc (Zn)
Superoxide dismutase [Fe]	Selenium (Se)
Vitamin B12 import ATP-binding protein BtuD	Nickel (Ni)
Copper resistance protein C	Copper (Cu)
Copper resistance protein B	Copper (Cu)
Copper resistance protein A	Copper (Cu),Silver (Ag)
Multidrug resistance protein MdtA	Zinc (Zn)
Multidrug resistance protein MdtB	Zinc (Zn)
Multidrug resistance protein MdtC	Zinc (Zn)
Outer membrane protein OprM	Copper (Cu),Silver (Ag)
HTH-type transcriptional regulator AdhR	Copper (Cu)

Trehalose transport system permease protein SugB	Molybdenum (Mo),Tungsten (W)
Staphyloferrin B transporter	Cobalt (Co),Zinc (Zn)
Phosphate regulon transcriptional regulatory protein PhoB	Zinc (Zn)
7-cyano-7-deazaguanine synthase	Aluminium (Al)
Copper-exporting P-type ATPase	Copper (Cu)
HTH-type transcriptional regulator CueR	Copper (Cu)
Lipoprotein-releasing system ATP-binding protein LolD	Tungsten (W)
Methionine import ATP-binding protein MetN	Iron (Fe)
Transcriptional regulatory protein ZraR	Zinc (Zn)
Formate hydrogenlyase transcriptional activator	Copper (Cu)
Hydrogenase-4 component A	Arsenic (As)
Iron-sulfur protein	Arsenic (As)
Formate dehydrogenase H	Arsenic (As)
High-affinity nickel transport protein	Nickel (Ni)
Apolipoprotein N-acyltransferase	Copper (Cu)
Magnesium and cobalt efflux protein CorC	Cobalt (Co),Magnesium (Mg)
Ferric uptake regulation protein	Iron (Fe)
KDP operon transcriptional regulatory protein KdpE	Zinc (Zn)
Potassium-transporting ATPase ATP-binding subunit	Cobalt (Co)
Zinc transporter ZitB	Zinc (Zn)
DNA-binding transcriptional dual regulator ModE	Molybdenum (Mo),Tungsten (W)
Molybdenum transport system permease protein ModB	Molybdenum (Mo),Tungsten (W)
Vitamin B12 import ATP-binding protein BtuD	Molybdenum (Mo),Tungsten (W)
High-affinity branched-chain amino acid transport ATP-binding protein LivF	Tungsten (W)
DNA protection during starvation protein	Iron (Fe)
putative ABC transporter ATP-binding protein YbiT	Iron (Fe)
Glutathione-binding protein GsiB	Nickel (Ni)

Glutathione transport system permease protein GsiC	Nickel (Ni)
Ferric-anguibactin receptor FatA	Cadmium (Cd),Cobalt (Co),Copper (Cu),Gallium (Ga),Iron (Fe),Manganese (Mn),Nickel (Ni),Zinc (Zn)
putative protein	Chromium (Cr)
Aquaporin Z	Antimony (Sb),Arsenic (As)
ATP-binding/permease protein CydD	Iron (Fe)
Thioredoxin reductase	Arsenic (As)
Cation/acetate symporter ActP	Tellurium (Te)
HTH-type transcriptional repressor ComR	Copper (Cu)
Nif-specific regulatory protein	Zinc (Zn)
Inner membrane ABC transporter permease protein YdcV	Molybdenum (Mo),Tungsten (W)
Spermidine/putrescine transport system permease protein PotB	Molybdenum (Mo),Tungsten (W)
Spermidine/putrescine import ATP-binding protein PotA	Molybdenum (Mo),Tungsten (W)

Fig S1: Circular genome of *Mangrovibacter* sp. SLW1

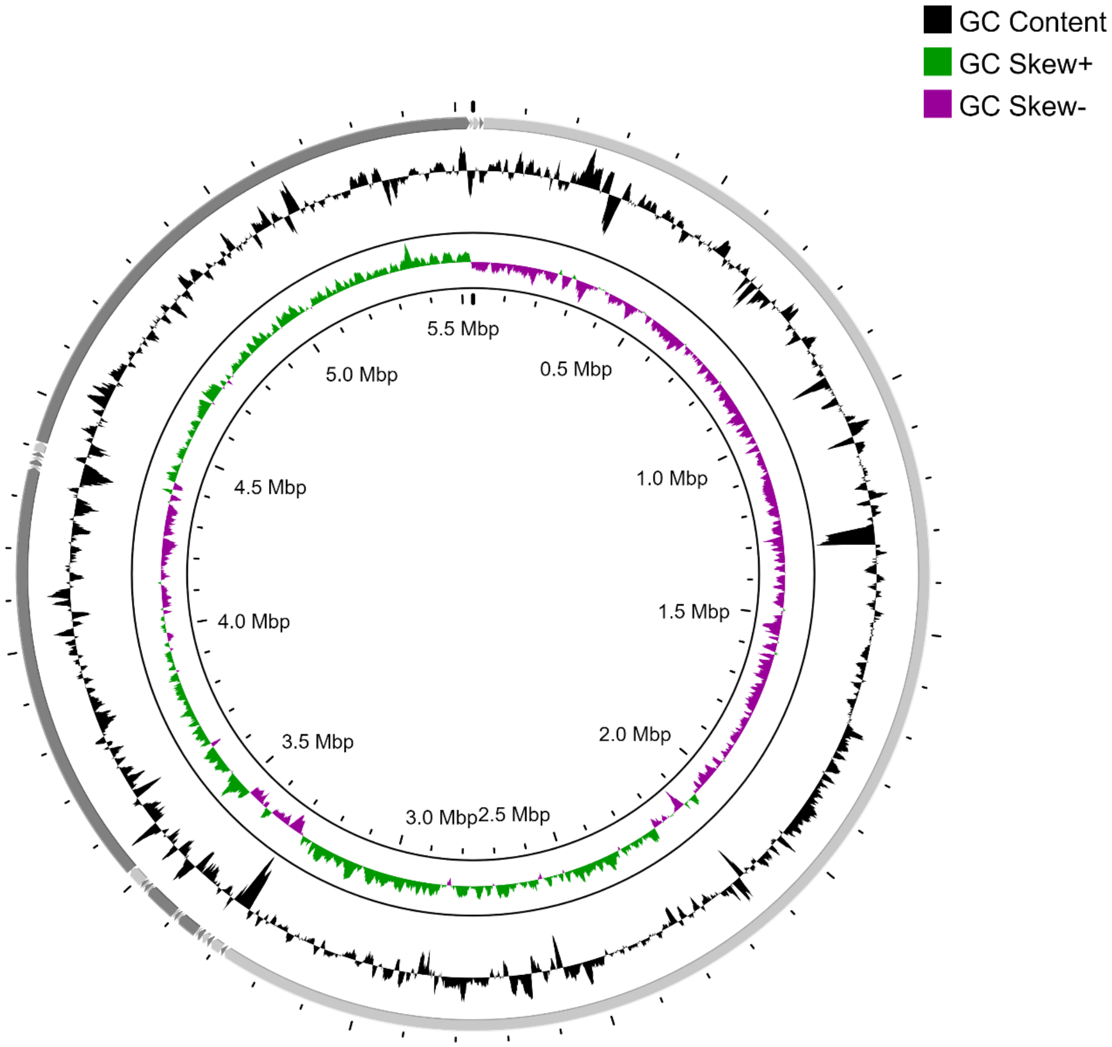

Fig S2: Phylogenomic position of *Mangrovibacter* sp. SLW1 derived from whole genome sequence

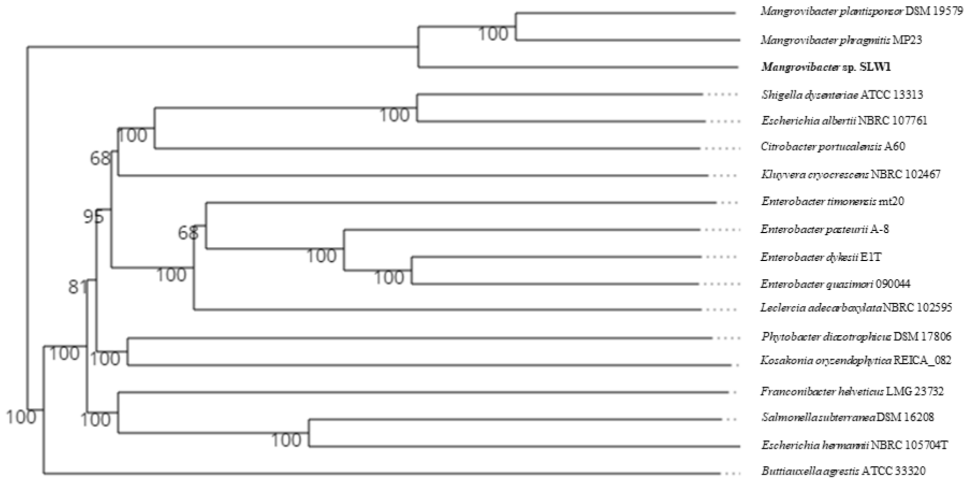

Supplement: Uncited Supplementary Material 1. [file acmi-6-00847-s001.pdf]
